# Chitosan modified with PAP as a promising delivery system for melatonin in the treatment of osteoporosis: targeting the divalent metal transporter 1

**DOI:** 10.1186/s13036-024-00422-7

**Published:** 2024-04-15

**Authors:** Weilin Zhang, Hongrui Rong, Jinguo Liang, Chao Mao, Zhencong Li, Zhiwen Dai, Dingbin Li, Weixiong Guo, Siyuan Chen, Zhongwei Wang, Jinsong Wei

**Affiliations:** Department of Spinal Degeneration and Deformity Surgery, Affiliated Hospital of Guangdong Medical University, Zhanjiang, 524001 China

**Keywords:** Osteoporosis, Melatonin, Chitosan

## Abstract

The demands for novel and efficient therapies have gradually increased with the rising concerns of osteoporosis (OP). The most popular method in promoting bone regeneration during osteoporotic conditions consists of loading bioactive materials with different drugs to treat osteoporotic bones by either promoting the process of osteogenesis, or by inhibiting the activity of osteoclasts. By analyzing single cell sequencing results, we found that divalent metal transporter 1 (DMT1) played a role in OP. Based on our previous results, we found that melatonin (MT) suppressed expression of DMT1 induced by high glucose during OP, so we determined the efficacy of MT for the treatment of OP. However, the clinical effects of MT on OP were unsatisfactory. To enhance its biological efficacy, we combined MT with porous gelatin chitosan (chitosan) and the conductive material, PLA-b-AP-b-PLA (PAP), then determined how MT incorporation in chitosan@PAP nanoparticles affected the ability to promote MC3T3-E1 osteogenesis and mineralization, both in vitro and in vivo. The results confirmed the effect of MT on DMT1. We then prepared and characterized composites prepared as nanofibers, and determined the efficacy of MT combined with chitosan-PAP modified hydrogels as a slow-release system in a femur model of osteoporosis mice, with associated properties suitable for bone tissue engineering. The results indicated that MT-loaded chitosan@PAP nanospheres showed favorable osteogenic functions, both in vivo and in vitro, providing a practical solution for bone regeneration for OP patients.

**Supplementary Information:**

The online version contains supplementary material available at 10.1186/s13036-024-00422-7.

## Background

Osteoporosis (OP) is a metabolic disease caused by imbalances between bone formation and absorption [1], which increases the fragility of bones. OP results in a high risk of bone fractures [2], which are the most severe complications of OP, and which may even lead to death [3]. Previous studies have reported that there were more than 8.9 million fractures annually and the morbidity of OP is still continuing to rise [4]. A report predicted that the number of OP patients will increase from 1.66 million in 1990 to 6.26 million by 2050 [5]. OP is therefore one of the major public health issues, which results in social, economic, and medical consequences. There is an urgent need to identify effective treatments for OP [6].

Anti-osteoporosis drugs comprise the common clinical treatments for OP. Melatonin (MT), discovered by Lerner in 1958 [7], is mainly secreted by the pineal gland [8]. MT is also synthesized and secreted by bone marrow tissue, especially in the femoral bone marrow [9]. MT is a potential anti-OP drug that could possibly suppress bone loss and promote new bone formation [10]. MT significantly reduced the expression of the divalent metal transporter 1 (DMT1) and improved the osteogenic capacity of osteoblasts by activating the Nrf2/HO-1 pathway, both in vivo and in vitro. Altered expression of the DMT1 gene has been found to affect OP in different ways [11]. For example, differentiation of osteoblasts can be affected by altering iron death metabolism [12]. DMT1 enrichment in mineral uptake pathways has also been found to be causally associated with the development of OP, in large-scale genetic analyses [13]. Based on previous reports, we speculated that MT may be an effective treatment for OP. However, the main limitations of using MT for the treatment of OP includes its low effective concentration, caused by its instability [14, 15]. Furthermore, MT is released too quickly in the body, so the treatment duration is insufficient [16].

To overcome these limitations, a suitable drug delivery system for slow-release of MT is warranted [17]. This approach may reduce the side effects and solve the insufficient sustainability issues. Hydrogels are three-dimensional polymer networks with high water content and with favorable abilities for drug loading and degradability, which are currently being used in a new generation of disease treatments [18]. This is especially true for injectable biodegradable hydrogels, which can reduce the dose and extend the therapeutic effects [19, 20]. Furthermore, injectable biodegradable hydrogels forming gels in situ are now being widely used in drug delivery [21]. Chitosan hydrogels, a common synthetic bone repair material, is used in drug loading because of its suitable biological properties and variable physical characteristics [22, 23]. Chitosan owns well manufacturability, mechanical properties, release kinetics, and biocompatibility and it has the potential to promote bone differentiation [24, 25]. However, the self-healing efficiency of pure hydrogels is limited, and this efficiency can be enhanced when combined with nanocomposites [26]. The triblock copolymer polylactide (PLA)-b-aniline pentamer (AP)-b-PLA (PAP) was synthesized by coupling an electroactive carboxyl-capped AP with two biodegradable bi-hydroxyl-capped PLAs via a condensation reaction. It has the unique properties oof being electroactive and biodegradable. We therefore combined chitosan with PAP to produce a new conductive nanocomposite-PAP and reported its use for the first time in bone tissue engineering. We further determined the therapeutic efficacy of MT loaded on the slow-release system of modified chitosan@PAP in a femur model of OP mice. This could be a new strategy for the treatment of OP patients.

## Materials and methods

### Data collection and preprocessing

We investigated the development of DMT1 in osteoporotic and non-osteoporotic bone marrow mesenchymal stem cell samples by analyzing human osteoporotic and non-osteoporotic bone marrow mesenchymal stem cell samples in an online database. Data from GSE147287 were downloaded from the Gene Expression Omnibus database. This database included single cell sequencing of osteoporosis (GSE147287) and osteoarthritis tissues (GSM4423511). Further down-clustering analysis, selection of differentially expressed genes, differential analysis, and acquisition of marker genes were performed using R software (version 4.2.0: R Foundation for Statistical Computing, Vienna, Austria) and its Seurat package (version 4.0) single cell data analysis software set developed by Satija Laboratories (New York, USA). Using this method, DMT1 was found to have potential in the development of osteoporosis, and pseudotime analysis revealed up-regulation of this gene expression in cells that developed OP.

### Pseudo-time analysis

Single cell pseudo-time analysis using Monocle2 (http://cole-trapnelllab.github.io/monocle-release) with DDR-Tree and its default parameter was used. Before monocle analysis, marker genes of Seurat clustering results and raw expression counts of the cells that passed filtering were selected. Based on pseudo-time analysis, branch expression analysis modeling was used for branch fate-determined gene analysis.

### Cell culture

The MC3T3-E1 cell line derived from mice (iCell, Shanghai, China) was cultured in α-MEM (Wisent, China) supplemented with 10% fetal bovine serum (FBS) and incubated at 37℃ in a 5% CO_2_ incubator. Before seeding, MC3T3-E1 cells were cultured and transferred using MEM medium supplemented with 10% FBS (Gibco, Grand Island, NY, USA) and 100 µ/mL penicillin-streptomycin-amphotericin B (Biotech, China), with daily fresh medium replacements.

### Cell viability assay

A cell viability assay was used to determine the cytotoxicity of MT (Sigma Aldrich, Shanghai, China) using the CCK-8 method. MC3T3-E1 cells were placed in 96-well plates at a density of 1 × 10^4^/well. They were then treated with varying amounts of MT (0, 5, 10, 25, 50, and 100 µmol/L) for 3 days. Then, 10 µL of CCK-8 cell viability buffer was added to each well, followed by incubation at 37℃ for an additional 1 h. Finally, the absorbance at 450 nm was determined using a microplate reader.

### Preparation of PAP, chitosan, and chitosan@PAP

Materials-chitosan was from Guangzhou Yunmei Technology (Guangzhou, China). The amino groups of p-phenylenediamine and N-phenyl-1,4-phenylenediamine, which was shielded with butane diacid anhydride, were dissolved in a solution containing dimethylformamide and HCl. Toluene (10 mM) was used to azeotropically distill the hydroxyl-capped PLA. Next, a flame-dried glass reactor was filled with 2 mmol of purified PLA, 1 mmol of emeraldine carboxyl-capped aniline pentamer (EMAP), 5 mmol of DCC, 5 mmol of DMAP, and 15 mL of NMP. Following the reaction, filtration was used to eliminate dicyclohexylurea. The copolymer present in the filtrate was precipitated using ethanol and subsequently dissolved in CHCl_3_. PAP was then dried at room temperature under vacuum. To prepare the chitosan Complex Coated PAP granules, ethyl acetate was initially used to disperse PAP (10 g). Water was then added to agglomerate the dispersed particle mixtures, which were then agitated. After the separation process, the resulting particles were dried. The granules that were screened were dispersed in the chitosan solution and subsequently coated with chitosan. Following 30 min of stirring, the granules that had been coated were isolated, rinsed with water, and subsequently dried in a desiccator. Sieve analysis was used to determine the size distribution of the desiccated granules.

### Cytotoxicity assessments of chitosan, PAP, and chitosan@PAP

Cell viability was assessed using the CCK-8 assay (Biosharp, Hefei, China) as per the instructions provided by the manufacturer to confirm the cytotoxic effects of chitosan, PAP, and chitosan@PAP. Briefly, the MC3T3-E1 cells were placed in a 96-well dish at a concentration of 3 × 10^4^ cells/mL and left to incubate for a period of 1 d. The MC3T3E1 cells were then exposed to chitosan, PAP, or chitosan@PAP in a culture medium for a duration of 3 d. Subsequently, 10 µL of CCK-8 was added to the culture and incubated at 37 °C in the absence of light for 3 h. A microplate reader was then used to measure the absorbance at 450 nm.

### Quantitative RT-PCR

MC3T3-E1 cells were cultured in 6-well plates with medium at a density of 10^5^ cells per well. The cells were subjected to treatment with MT at concentrations of 0, 5, 10, 25, 50, and 100 µmol/L. After 3 d, the RNA from cells was obtained using a rapid extraction kit designed specifically for RNA. Subsequently, the RNA was converted into cDNA using a reverse transcription kit (Beyotime, Shanghai, China). Next, SYBR Green PCR Master Mix (Thermo Fisher Scientific, Waltham, MA, USA) was used to conduct real-time quantitative PCR. To ensure data accuracy, 40 cycles of PCR were performed with the following conditions: 10 min at 94℃, 15 s at 95℃, and 60 s at 60℃. In addition, six replicate wells were used for all reactions.

### In vitro release of chitosan@MT and chitosan@MT/PAP

After the gel formed, 100 µL of a liquid solution with a concentration of 50 μm/L of MT were added to chitosan/chitosan@PAP and stirred, shaked for 2 h, then placed in a 24-well plate with 500 µL of phosphate-buffered saline (PBS). The well plates were placed in a shaking incubator at 100 rpm and incubated for 14 d at 37 °C. The supernatant was collected with fresh PBS (0.1 M) at specific intervals [0 day, 1 day, 2 days, 3 days, 4 days, 5 days, 6 days, 7 days (1 w), 10 days, and 14 days (2 w)]. The supernatants were then preserved at -80 °C until ready for the ELISA test. The concentration of MT in each sample was evaluated using a Human MT ELISA Kit (Fine Test, China) following the manufacturer’s instructions, at a wavelength of 450 nm. The measurement was conducted six times for each time period, and the total quantity of MT was computed and graphed over time.

### Microparticle characterization

The morphology of microparticles was determined using scanning electron microscopy (SEM; Gemini 2, Zeiss, Oberkochen, Germany). To prepare the samples, a small amount of the microparticle mixture was placed on the surface and left to dry for an entire night. The microparticles were then analyzed using INVENIO-R spectrometer Fourier transform infrared spectroscopy (FTIR; Bruker, Billerica, MA, USA). The dried hydrogels were scanned using a Micro CT (PE Quantum GX2; Perkin Elmer, Waltham MA, USA) and the xx software was used to calculate the percentage of total porosity.

### Immunohistochemistry analysis

Immunohistochemistry was used for detection of RUNX-2 proteins. The MC3T3-E1 cells were cultured in a 6-well plate at a density of 10^4^ cells per well. They were then divided into four groups: Control, chitosan, chitosan@PAP, chitosan@MT, and chitosan@MT/PAP to co-cultured. Following a 1-week co-culture period, MC3T3-E1 cells were placed on medium at a concentration of 5 × 10^5^ cells/mL. Next, the medium was supplemented with osteogenic induction medium and incubated for a duration of 48 h. Following fixation of cells in 4% paraformaldehyde for 30 min, the slides were treated with goat serum to prevent nonspecific binding. Subsequently, they were incubated with primary antibody (antiRUNX-2, 1:200; Abcam, Shanghai, China) overnight at 4ºC. Next, the medium was treated with RUNX-2 conjugated secondary antibody (at a dilution ratio of 1:50; source and address) for 30 min at ambient temperature. Then, images were captured using a microscope.

### Immunofluorescence staining

The MC3T3-E1 cells were cultured in a 6-well plate at a density of 10^4^ cells per well. They were then divided into five groups: Control, chitosan, chitosan@PAP, chitosan@MT, and chitosan@MT/PAP for co-culture. Following 1 week, the MC3T3-E1 cells were rinsed using PBS, then treated with 4% paraformaldehyde for 30 min, and subsequently permeabilized with Triton X100 for 10 min. Next, the cells were cultured with primary anti-RUNX-2 antibody at a temperature of 4℃ for the entire night. Afterward, they were stained with anti-RUNX2 and Alexa Fluor 488 Phalloidin (Molecular Probes, Eugene, OR, USA) in the absence of light for 1 h. Following a 10 min fixation period using mounting fluid containing 4’,6-diamidino-2-phenylindole, the images of cells were captured using the EVOS M5000 cell imaging system (Thermo Fisher Scientific).

### Establishing an osteoporosis mouse model and in vivo experiments

A total of 32 C57BL/6 female mice, 8-weeks-old and weighing approximately 20 g each, were purchased from Ruige (Shenzhen, China). These mice were then randomly assigned to four groups: Control group, chitosan@PAP group, MT group, and chitosan@MT/PAP group. After anesthesia, all mice underwent surgery, and the incisions were closed following the removal of both ovaries and the surrounding adipose tissues. After a period of 4 weeks, the chitosan@PAP/MT group received a single injection of 10 mg/kg chitosan@MT/PAP. The chitosan@PAP group was injected with the same dose as the chitosan@MT/PAP group. Similarly, the MT group received the same dose of MT, and the control group received the same dose of saline. Following a 4-week intervention, mice were sacrificed and the femur was extracted and immobilized in a 4% paraformaldehyde solution. The mouse femur in PBS at room temperature were used to observe the bone microstructure. The femur bones underwent hematoxylin & eosin (HE) staining and micro-computed tomography (CT) analyses. Micro-CT was utilized for quantitative determination in both two-dimensional (2D) and three-dimensional (3D) formats, following previously established protocols [27–29]. The Siemens Preclinical Imaging System (Siemens, Amsterdam, The Netherlands) was used to measure microstructure parameters of cancellous bone, including trabecular number (Tb.N), trabecular thickness (Tb.Th), bone mineral density (BMD), and bone volume/total volume (BV/TV). These measurements were conducted using the multislice mode in standard resolution.

### Statistical analysis

The mean ± standard deviation of quantitative data obtained from a minimum of six biological replicates is presented in this study. Statistical significance was evaluated using SPSS 20.0 statistical software for Windows (SPSS, Chicago, IL, USA). Student’s *t*-test and analysis of variance were used to determine the distinctions between 2 and > 2 groups. Groups were considered significantly different at a value of *P* < 0.05.

## Results

### Single cell sequencing analysis

**Fig. 1:**
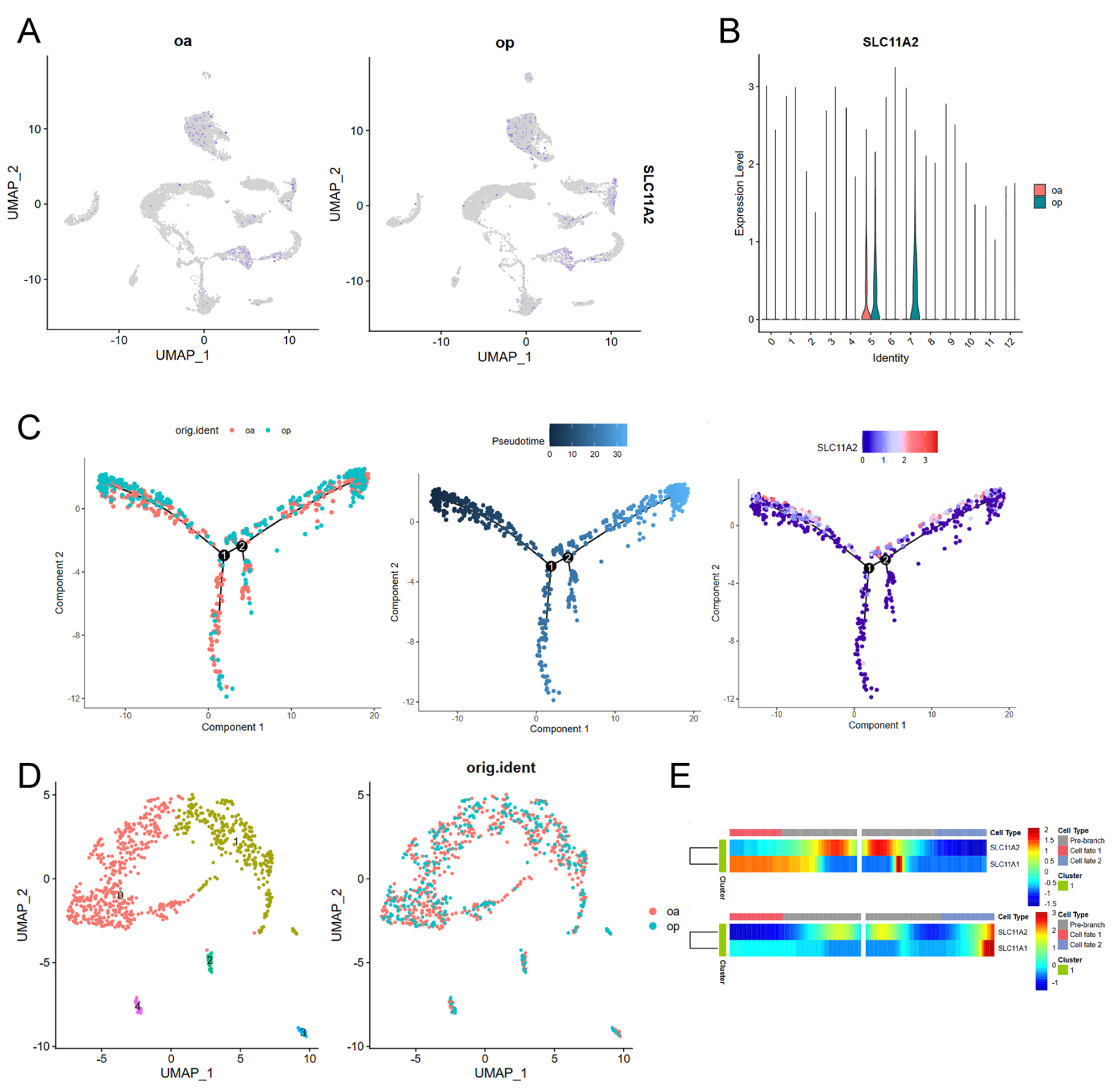
Single cell sequencing analysis of osteoporosis (OP) and nonosteoporosis samples. (**A**). Uniform Manifold Approximation and Projection(UMAP ) plots show the expression distribution of DMT1 in osteoporosis (OP) and osteoarthritis (OA)samples from the GSE147287 database. (**B**). Violin diagrams are presented to compare the expressions of DMT1 in OP and OA samples from the GSE147287 database. (**C**). Pseudo-time showing the progression of differentiation of OP and OA cells, and changes in DMT1 expression during differentiation progression. (**D**). UMAP profiles show the distribution of cell cluster 5 in OA and OP samples. (**E**). Heat map showing changes in expressions of DMT1 for pre-branch and cell fate using a quasi-time series analysis

We investigated the development of DMT1 in osteoporotic and nonosteoporotic bone marrow mesenchymal stem cell samples by analyzing human osteoporotic and non-osteoporotic bone marrow mesenchymal stem cell samples using an online database. DMT1 was found to be slightly more highly expressed in osteoporotic samples, by analyzing violin plots of gene expressions in both databases (Figure 1B). However, it was not evident in the dimensionality reduction display of genes (Figure A). Subsequently, we performed a dimensionality reduction analysis (Figure 1D) and cell differentiation pseudo-time analysis (Figure 1C) of cell cluster 5 in two samples, which revealed that cells from osteoporotic samples were enriched in the tail end of the trajectory, suggesting that osteoporotic cells involved differentiation of nonosteoporotic cells. Genes in this differentiation trajectory showed that DMT1 showed no significant trend in the distribution of DMT1 in the differentiation trajectory, but upregulation of DMT1 was observed at the end of the cell fate in the pseudo-time analysis heat map (Figure 1D). DMT1 was found to have some regularities in the development of osteoporosis using this analysis, and pseudo-time analysis revealed up-regulation of this gene expression in cells developing OP.

### MT treatment depresses the expression of DMT1 and promotes the osteogenic differentiation of MC3T3-E1 cells

**Fig. 2:**
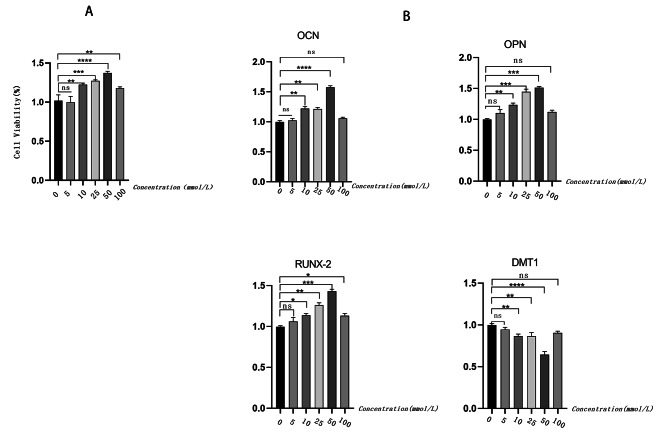
The effect of melatonin (MT) on cell viability and gene expression. (A). Three day CCK-8 cell viabilities determined after treatments with different concentrations of MT. (B). RT-PCR of samples treated with different concentrations of MT. *n* = 6 for each group. ^*^P < 0.05; ^**^P < 0.01; ^***^P < 0.001; ^****^P < 0.0001

To determine the relationship between DMT1 and MT, we first validated the effects of MT treatment on MC3T3-E1 cells in vitro. The results confirmed that MT had no cytotoxicity on MC3T3-E1 cells. In contrast, MT promoted cell proliferation (Fig. 2A); we showed that the relative mRNA levels of three osteoblast marker genes, Alkaline phosphatase(ALP), RUNX Family Transcription Factor 2(RUNX2), and osteopontin (OPN), were significantly increased by MT treatment of MC3T3-E1 cells, while DMT1 depressed these effects (Fig. 2B), especially at a concentration of 50 µmol/L. Together, these results showed that MT enhanced MC3T3-E1 viability and promoted osteogenic differentiation without cytotoxic effects, and confirmed MT stimulated osteogenic differentiation of MC3T3-E1 cells by depression of DMT1 expression.

### Material characteristics

**Fig. 3:**
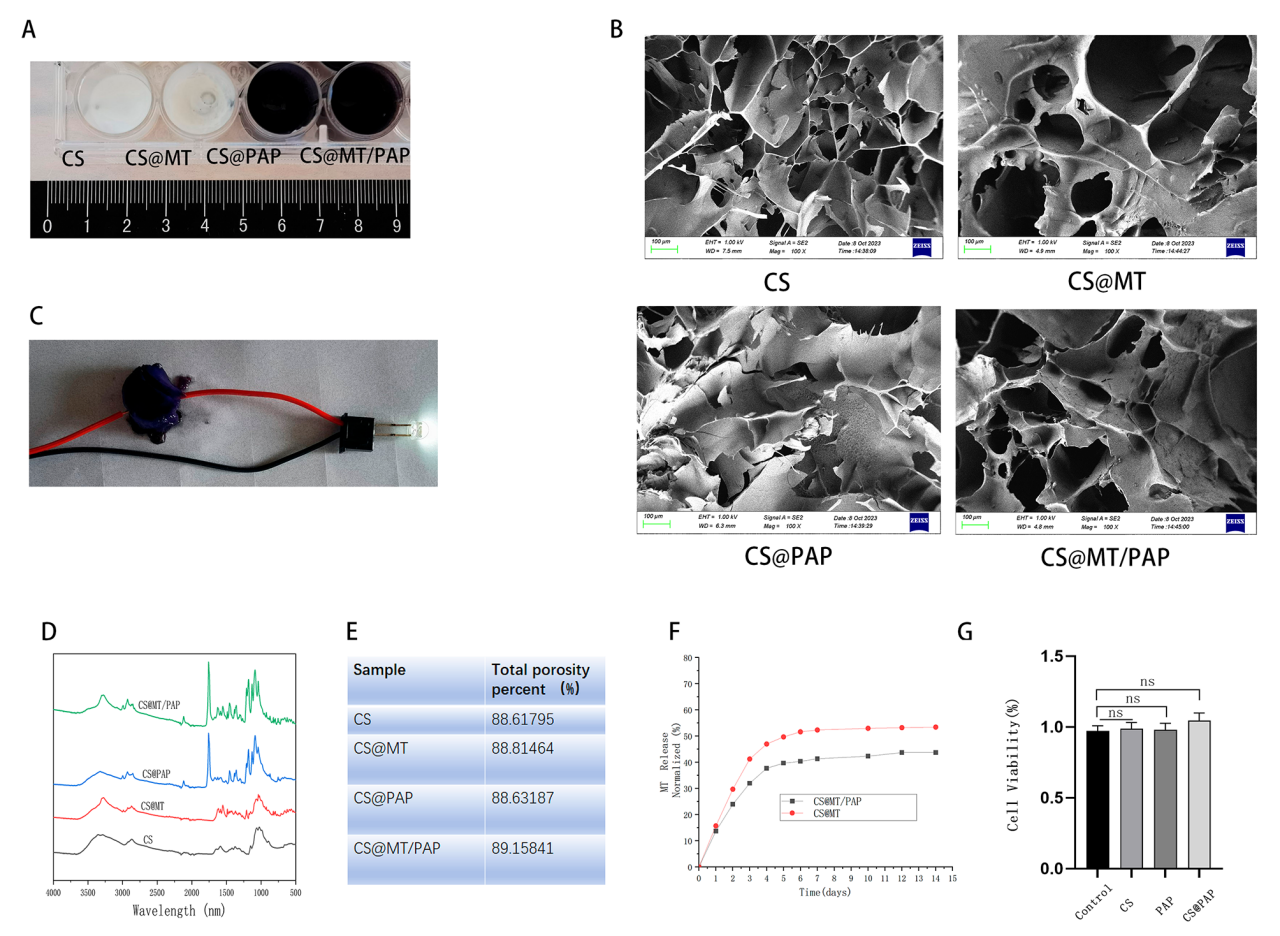
Material characteristics of chitosan, chitosan@MT/PAP, chitosan@MT, and chitosan@PAP, CS: chitosan. (**A**). External phase of chitosan, chitosan@MT/PAP, chitosan@MT, and chitosan@PAP. (**B**). Scanning electron microscopy of dried hydrogels: chitosan, chitosan@MT/PAP, chitosan@MT, and chitosan@PAP. (**C**). Electrical conductivity of chitosan@PAP. (**D**). Fourier transform infrared spectroscopy of chitosan, chitosan@MT/PAP, chitosan@MT, and chitosan@PAP. (**E**). Total porosity percentages of chitosan, chitosan@MT/PAP, chitosan@MT, and chitosan@PAP. (**F**). Time course of the in vitro release profile of MT (*n* = 6 for each group). (**G**) The 3-day CCK-8 cell viabilities of MC3T3-E1 cells co-cultured with chitosan, chitosan@MT/PAP, chitosan@MT, and chitosan@PAP. ^*^P < 0.05; ^**^P < 0.01; ^***^P < 0.001; ^****^P < 0.0001

After verifying the function of MT in promoting osteogenic differentiation, we developed chitosan and chitosan@PAP to construct a slow-release system for MT. The porous structures of chitosan, chitosan@MT/PAP, chitosan@MT, and chitosan@PAP were observed in the external phase (Fig. 3A), FTIR analysis (Fig. 3D), and SEM images (Fig. 3B). It showed that the hydrogels had interconnected porous structure and well fluidity and also showed that the chitosan hydrogels usually peak between 750 and 1250 nm while the PAP is between 1600 and 1900 nm. The total porosity percentage of chitosan@MT/PAP hydrogels (Fig. 3E) in the freeze-dried state was 89.15841%. Its high porosity percentage allowing for the continuous release of enclosed medications, cellular penetration, and intra-extra substance exchange within the hydrogels. Furthermore, chitosan@PAP exhibited remarkable metallic conductivity as depicted in Fig. 3C. It showed that the electroconductibility of PAP was not affected after being combined with chitosan and was being co-cultured with MC3T3-E1 for 3 days. In addition, the result showed that chitosan, chitosan, PAP and chitosan@PAP had no cytotoxic effects (Fig. 3G).

### In vitro releases of chitosan@MT and chitosan@MT/PAP

We then loaded MT into chitosan and chitosan@PAP and assayed the release speed of the slow-release system. The aim of embedding the MT in the chitosan and chitosan/PAP was to obtain a controlled release pattern over time. ELISAs were used to quantitatively measure MT that was released. The reliability of this method was evaluated to determine its effectiveness in monitoring the in vitro release of MT embedded with chitosan. The total amount of MT released in the supernatant was determined as a percentage of the initial chitosan-loaded quantity (100%). Using ELISAs, a value of 100% was equivalent to 1 µg of MT. Figure 3F shows an initial burst release of MT of 25% according to the ELISA assay. Between 5 d and 14 d (336 h), the ELISAs showed a steady and consistent release of the drug over time. Together, these results showed that chitosan@MT/PAP exhibited a superior slow-release capability, when compared with chitosan@MT.

### Functionality of the slow-release system during osteogenic differentiation

**Fig. 4:**
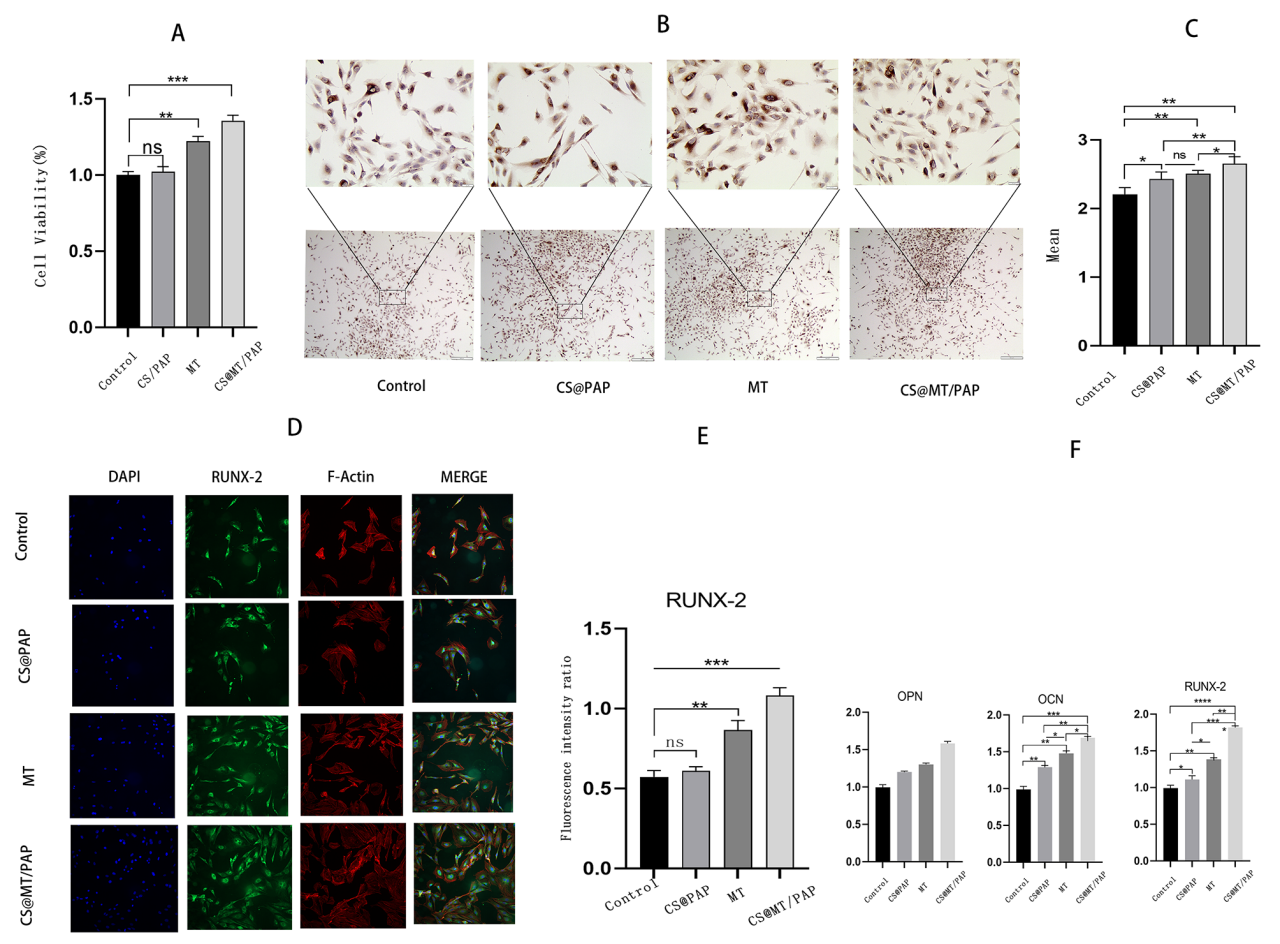
Effect of the slow-release system co-cultured in MC3T3-E1 cells, CS: chitosan (**A**). The 3-day CCK8 cell viability of MC3T3-E1 cells co-cultured with chitosan@PAP, MT, and chitosan@MT/PAP. (**B**). Immunostaining of MC3T3-E1 cells co-cultured with chitosan@PAP, PAP, and chitosan@MT/PAP. Boxed areas are shown at higher magnification on top. (**C**). Analysis of sepia areas. (**D**). Immunofluorescence staining of MC3T3-E1 cells co-cultured with chitosan@PAP, MT, and chitosan@MT/PAP. (**E**). Analysis of immunofluorescence staining. (**F**). RT-PCR of samples co-cultured with chitosan@PAP, MT, and chitosan@MT/PAP. *n* = 6 for each group. ^*^P < 0.05; ^**^P < 0.01; ^***^P < 0.001; ^****^P < 0.0001

Three-day CCK-8 viability assays of MC3T3-E1 cells co-cultured with chitosan, PAP, and chitosan@PAP showed that chitosan, PAP, and chitosan@PAP had no effect on proliferation of MC3T3-E1 cells (Fig. 4A). We assayed the MT slow-release system after 3 d for cell viability, immunostaining, and immunofluorescence, to determine the osteogenic ability of the slow-release system of MC3T3-E1 cells. The 3-day CCK8 cell viability assay of MC3T3-E1 cells co-cultured with chitosan@PAP, MT, and chitosan@MT/PAP showed that the MT group and chitosan@MT/PAP enhanced cell viability of MC3T3-E1 cells (Fig. 4D). Using immunostaining, uniformly distributed brown markers for RUNX2 were observed throughout the cytoplasm (Fig. 4B and C), and RUNX-2 was expressed in the cytoplasm. However, brown markers for RUNX-2 were observed less frequently in the control group, while RUNX-2 expression was frequently observed in other groups, especially in the chitosan@PAP /MT group. The results showed that MT enhanced the expression of RUNX-2, and that that RUNX-2 expression increased in the chitosan@PAP group. Immunofluorescence staining also showed that chitosan@MT/PAP significantly enhanced the expression of RUNX-2 (Fig. 4DE). In RT-PCR, we showed that the relative mRNA levels of three osteoblast marker genes, ALP, RUNX2 and OPN, were significantly increased in chitosan@MT/PAP group as well.

### The MT-Load slow-release system promotes OP mice femur bone formation

**Fig. 5:**
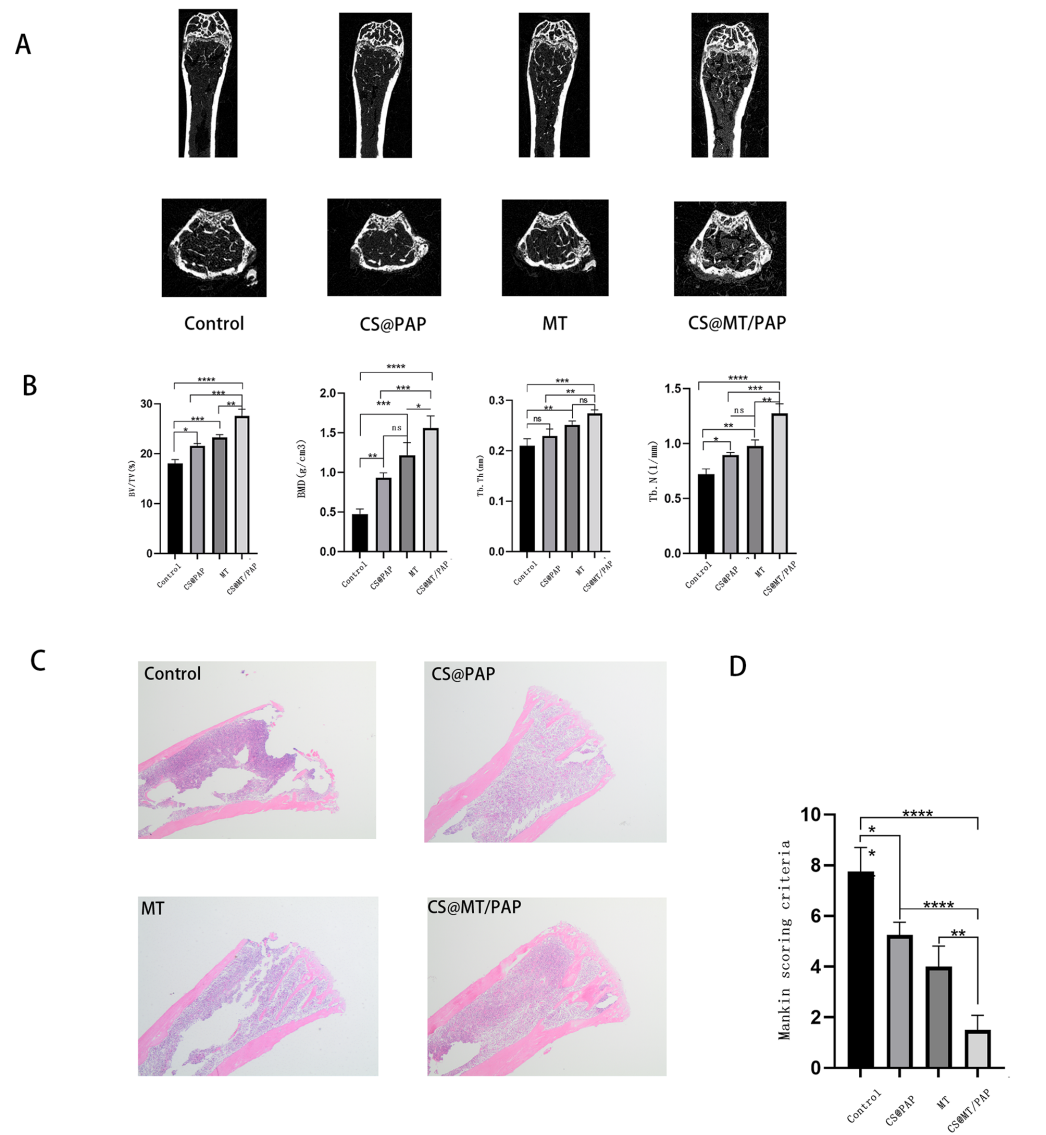
In vivo experiment result, CS: chitosan. (**A**). Representative micro-computed tomography (CT) images of the distal femurs at 4 w posttreatment. (**B**). Analysis of the distal femoral trabecular bone parameters using micro-CT. The micro-CT images of trabecular bone femurs after 4 w with comparative analyses of bone structural parameters, including bone volume/total volume (BV/TV), bone mineral density (in g/mm^3^, trabecular thickness (Tb. Th, in mm), and number of bone trabeculae (TB. N, in 1/mm). (**C**). Hemoxylin & eosin staining of femoral sections of mice at 4 w posttreatment. (**D**). Mankin’s criteria (femurs = 8 for each group). ^*^P < 0.05; ^**^P < 0.01; ^***^P < 0.001; ^****^P < 0.0001

Following the development of the OP mice model and a 4-week treatment period, mice that received chitosan@PAP, MT, or chitosan@PAP/MT exhibited elevated BMD values, when compared with OP model mice (Fig. 5AB). Furthermore, the number of TB.N and Tb.Th in the chitosan@PAP/MT group mice were observed to significantly increase (Fig. 5B), whereas the trabecular pattern factor showed a significant decrease, when compared with OP model mice. Femur sections from each group underwent H&E staining, and three physicians specializing in spine surgery evaluated the H&E staining based on the Mankin’s grading system (Supplementary Table 1) [27–29]. OP mice exhibited decreased trabeculae, especially in the metaphyseal region of the distal femur. The use of the chitosan@PAP and MT groups, either alone or in combination, resulted in an increase and thickening of the bone trabeculae, indicating promotion of new bone formation after treatment. The statistical findings indicated that significant bone deterioration in OP mice could be successfully corrected after treatment with chitosan@PAP/MT (Fig. 5CD). Overall, these results illustrated the osteogenic ability of the chitosan@MT/PAP release system.

## Discussion

Although previous studies have reported a direct association between MT and DMT1, the evidence is not conclusive. Comprehensive and multi-level, single cell sequencing accurately measures gene expression levels and identifies even small quantities of gene expressors or uncommon noncoding RNAs. The significant involvement of DMT1 in OP has been revealed through our preliminary findings from single cell sequencing analysis. DMT1 has recently emerged as a new focus for the prevention and management of OP [30]. Earlier research has shown that MT induced osteogenic differentiation by downregulating the expression of DMT1 [11]. Initially, we tested the non-toxicity of MT on MC3T3-E1 cells. Subsequently, we analyzed changes in osteoblast marker genes and DMT1 expression profiles in MC3T3-E1 cells after MT treatment, using RT-PCR analysis. These alterations were found to be linked to various biological processes. In MC3T3-E1 cells, MT was found to further suppress the expression of DMT1. The present results support these findings, showing that MT actually affected osteogenic differentiation.

We then synthesized PAP nanoparticles coated with chitosan and found a porosity of 88.63187%. Additionally, we showed that chitosan, PAP, and chitosan@PAP did not affect the proliferation of MC3T3-E1 cells. Subsequently, we devised a novel drug delivery system utilizing chitosan and chitosan@PAP to load MT. The results showed that chitosan@MT/PAP exhibited superior slow-release capabilities, when compared with chitosan@MT. Finally, we chose the best concentration (50 µmol/L) of MT that stimulated osteogenesis, to construct a chitosan@MT/PAP release system, to determine its osteogenic ability. The viability of MC3T3-E1 cells was assessed using CCK-8 assays, and the results from immunofluorescence staining showed that chitosan@MT/PAP greatly improved cell viability and osteogenic activity. However, the mechanism responsible for the significant enhancement of MC3T3-E1 cell viability and osteogenic ability by chitosan@MT/PAP remains unknown.

After confirming the effectiveness of the MT slow-release system using in vitro experiments, we conducted subsequent in vivo experiments. The results showed that chitosan@MT/PAP administration resulted in faster growth and better bone regeneration in the femurs of OP mice, when compared with the chitosan and MT groups, especially at 4 w. To our surprise, chitosan@PAP alone without melatonin already demonstrated pretty good osteogenic property, we speculate that the substantial ratio of surface area to volume and the level of porosity in chitosan/PAP contributed to the favorable adhesion of osteoblasts and the gradual release of MT. Meanwhile, the remarkable metallic conductivity of chitosan@MT/PAP may have the potential to promote osteogenic differentiation by transfer ing bioelectricity between osteoblast, but the mechanism is not clear yet.

Previous research used MT for the treatment of osteoporosis through intraperitoneal delivery [31, 32], resulting in decreased bone loss. However, longtime intraperitoneal injection may result in bodily injury and even infection of OP patients. When compared with intraperitoneal injections, the slow-release system of one subcutaneous injection decreased the potential for invasive injury. A previous study also prepared MT-loaded chitosan microparticles and showed that MT promoted the differentiation and mineralization in preosteoblast cells [33]. Nevertheless, the absence of in vivo experiments raises uncertainty about the therapeutic efficacy in living organisms. In addition, the research showed that Mel-loaded CS MPs have significantly enhanced alkaline phosphatase (Alp) mRNA expression and ALP activity. However, MT-loaded chitosan microparticle fabricated by ionic crosslinking method soon released about 50% MT in 2 days, although in vitro experiment result confirmed the osteogenic capability, the in vivo experiment domonstrated the drug may not unable to continue releasing. By incorporating the conductive substance PAP, our study aimed to improve the flexibility and effectiveness of pure hydrogels. In addition, we conducted an in vivo experiment using the chitosan@PAP/MT slow-release systems, which showed the benefits of an injectable scaffold. In other reports, injectable scaffold always showed an increased number of osteoblast cells, as well as their proliferation, which may due to the porous structure allowing cell living [34]. However, the number of osteoblast cells and proliferation showed in CS@PAP was a little out of ordinary compared to non-conducting materials. One possible explanation for this phenomenon was that the hydrogel injection created a more compatible connection with the bone, facilitating the transfer of MT to the implant. In addition, it may have enhanced stress transmission, leading to increased bone generation, when compared with conventional intravenous and intraperitoneal methods.

## Conclusions

Biological Engineering is a comprehensive use of mathematics, physics, chemistry, biology knowledge, as well as the method of engineering itself, in order to deal with various problems in the fields of biology and medicine, etc., to meet the human needs for biological products of an engineering. As a biological engineering research, we artificially rebuilt healthy bone by using nanocomposite and molecular biology, to meet the problems of osteoporosis in the medical field. In conclusion, we further confirmed the osteogenic ability, and verified MT as an efficient drug against OP, which provided evidence for the possible clinical use of MT for OP patients. Furthermore, we showed the effects of MT-loaded chitosan@PAP nanospheres on mice with osteoporosis. The findings indicated that chitosan@MT/PAP was a highly effective and user-friendly method for delivering MT and for promoting bone regeneration. chitosan@MT/PAP served as a scaffold to target a specific region of bones, and the gradual release of drugs to treat OP. This approach eliminates the need for multiple injections, and provides a straightforward way to regulate drug release. The chitosan@MT/PAP nanoparticles can therefore be used for in vivo treatment of osteoporosis. For the first time, chitosan@MT/PAP scaffolds have been created as therapeutic bone scaffolds that included PAP as an innovative nanocomposite to improve local bone regeneration. The slow-release mechanism was a successful method for releasing MT and a suitable framework for tissue engineering, providing a practical solution for bone regeneration for OP patients.

### Electronic supplementary material

Below is the link to the electronic supplementary material.

Supplementary Material 1

## Data Availability

The raw data of transcriptome sequencing are available at GEO Data Sets of NCBI (GSM4423511 and GSE147287).
